# The Immunomodulatory Activity of High Doses of Vitamin D in Critical Care Patients with Severe SARS-CoV-2 Pneumonia—A Randomized Controlled Trial

**DOI:** 10.3390/nu17030540

**Published:** 2025-01-31

**Authors:** Ana Moura Gonçalves, Sónia Velho, Bárbara Rodrigues, Maria Lobo Antunes, Miguel Cardoso, Ana Godinho-Santos, João Gonçalves, António Marinho

**Affiliations:** 1 Intensive Care Medicine Department, Hospital Beatriz Ângelo, 2674-514 Loures, Portugal; 2 Nutrition Department, Hospital Beatriz Ângelo, 2674-514 Loures, Portugal; 3 Faculty of Pharmacy, iMed—Research Institute of Medicines, University of Lisbon, 1649-004 Lisbon, Portugal; 4 Faculty of Medicine, Instituto de Ciências Biomédicas Abel Salazar, University of Porto, 4099-002 Porto, Portugal

**Keywords:** SARS-CoV-2 pneumonia, vitamin D, cholecalciferol, vitamin D receptor [VDR], LL-37, cathelicidin, polyvalent intensive care unit

## Abstract

Vitamin D receptor [VDR] expression promotes LL37 expression, possibly contributing to host defense. The hypothesis was that an increase in 25 hydroxyvitamin D [25vitD] could lead to enhanced VDR expression and increased LL-37 production, thereby contributing to improved prognosis in critically ill patients. **Methods**: A nonblinded, randomized controlled trial was conducted. A total of 207 patients admitted to ICU with severe SARS-CoV-2 pneumonia were included and received different doses of cholecalciferol (500 MU, 3 MU/day, no cholecalciferol) during their ICU and hospital stay. 25vitD levels as well as LL37 and monocytes’ VDR gene expression were evaluated on admission and after. Clinical evolution, ICU mortality, hospital mortality, and 60-day mortality were evaluated. **Results:** The median age was 57.7 years and the majority of patients were Caucasian [87.4%] and male [70.5%]. There was a significant difference in 25vitD levels between groups on the third [*p* = 0.002] and seventh [*p* < 0.001] days. Patients supplemented with 500 MU of cholecalciferol had a very significant increase in monocytes’ VDR gene expression and showed a better clinical evolution in the ICU, with a significant correlation to evolution factors. Higher LL37 on admission had a significant negative association with hospital and ICU mortality, lost after adjustment for comorbidities to a nearly significant association with ICU, hospital, and 60-day mortality. **Conclusion**: Supplementation with higher doses of cholecalciferol may contribute to a significant increase in 25vitD levels but not in LL37 levels. Higher LL37 levels on admission may be related to a decrease in ICU, hospital, and 60-day mortality. VDR gene expression in monocytes is much higher in patients supplemented with higher doses of cholecalciferol.

## 1. Introduction

Vitamin D (vitD) has known anti-inflammatory [1] and immunomodulatory activities [2,3] and low vitD levels are associated with an increased rate of respiratory tract infections in children and adults [4,5,6], with a worse prognosis in these patients, longer disease duration, and greater complications [7,8].

Vitamin D levels are decreased in critically ill patients [9,10,11,12,13,14], with lower levels being linked to increased sepsis [15], acute respiratory distress syndrome, increased length of stay [16], acute kidney injury [17], and mortality [10,16].

Due to its receptor (VDR), present in most body tissues, vitD modulates the innate and acquired immune system [3,18,19] and stimulates reactive oxygen species and antimicrobial peptides (AMPs), cathelicidins, and defensins [20]. Macrophages are cells of the immune system specialized in innate defense against pathogens [21]. Besides inducing cathelicidin [19], vitD enhances the differentiation of macrophages from monocytes [22], as well as their activation [22], autophagy, and phagosome maturation [22,23]. Lung epithelial cells, in the presence of a viral infection, are able to convert inactive vitamin D, 25vitD, into its active form, 1.25-dihydroxycholecalciferol (1.25vitD), also producing an increase in cathelicidin [20,24], which plays an important role in the immunological defense against viruses [25], Gram-positive and Gram-negative bacterial agents [26,27,28,29], and fungi [30]. Cathelicidins interact with pathogen cellular membranes, causing their instability, promoting membrane rupture and cellular destruction, and leading to pathogen death [29].

Leucine-leucine-37 (LL-37), the only human cathelicidin, is cleaved from its precursor hCAP18 [31] and has protective functions in the human body. It is an antimicrobial peptide, and vitD is able to upregulate its production [19]. LL-37 has antibacterial, antiviral, and antifungal activity [32,33,34], as it disrupts bacterial membranes through electrostatic interactions [35] and promotes the destruction of viral wall proteins [36]. There is some evidence supporting vitD induction of LL-37 production by monocyte-derived macrophages and LL37’s contribution to host defense [37].

Studies have linked vitD deficiency in critical care patients to worse outcomes, including higher severity of illness, longer ICU stays, prolonged mechanical ventilation, greater rates of infections and organ dysfunction, and increased mortality risk. These patients also incur higher ICU and hospital costs [38,39,40,41,42,43]. A review of 14 studies with more than 9000 critically ill patients found vit D deficiency was associated with higher risks of sepsis, septic shock, and death [44]

The effects of vitamin D supplementation in ICU patients have been evaluated in several studies [45,46,47,48,49].

Oral cholecalciferol at doses ranging from 200 to 540,000 IU was administered as single or multiple doses. No clinically significant adverse effects were reported in any of these studies. The only observed outcome was transient hypercalcemia, which did not require intervention [46]. In a randomized controlled trial (RCT) using high-dose vitD, only mild hypercalcemia was recorded with no serious adverse events [49].

Research on vitamin D supplementation in critically ill patients has yielded inconsistent findings, with some studies demonstrating beneficial effects, while others report no significant impact. Importantly, no evidence of harm associated with its use has been identified [49,50].

Critically ill patients represent a highly complex population, making it challenging to achieve absolute certainty in research studies due to the substantial heterogeneity in patient characteristics, interventions, and outcomes.

Severe SARS-CoV-2 pneumonia brought together ICU patients with serious clinical conditions, similar characteristics, and an undeniable inflammatory process, making them more easily comparable. Almost ideal conditions were met to study vitD in this particular population of ICU patients, assessing the effects of different doses on clinical evolution while analyzing LL-37 levels and VDR gene expression.

We theorized that maintaining higher levels of vitD through the administration of high doses of cholecalciferol could have an effect on VDR gene expression and LL37 levels and be beneficial to patients with severe SARS-CoV-2 pneumonia admitted to an intensive care unit.

However, some particularities need to be considered: there may be vitD resistance, malabsorption, or increased catabolism in critical care patients, which can hinder therapeutic efficacy. Resistance to cholecalciferol related to genetic abnormalities in vitD receptors was excluded since patients had no signs of rickets.

Because malabsorption and resistance to cholecalciferol related to age could exist in our patients [51], patients were split into two groups according to their response to supplementation. In critically ill patients, increased catabolism could also occur, leading to lower vitD levels, as well as interaction with other drugs, such as phenytoin, carbamazepine, isoniazid, theophylline, rifampin, and others, resulting in a P450 enzyme activity increase, metabolizing vitamin D into inactive metabolites [52,53,54].

VitD levels are low in critically ill patients [9,10] and rapidly fall after ICU admission [11] due to metabolism dysregulation. Evidence suggests that critically ill patients with low 25vitD levels may exhibit diminished responsiveness to vitD supplementation, potentially due to the conversion of vitD into alternative metabolites or epimers [55].

There are no reports of toxicity in this particular population supplemented with short-term high doses of cholecalciferol [48,49]. In our previous study, we concluded that patients supplemented with short-term higher doses of cholecalciferol (250 MU/day, for 2 days) had a significant increase in 25vitD levels during their ICU stay when compared to patients with lower doses (3 MU/day) or no supplementation [56].

The main objective of this study was to evaluate if the increase in 25vitD levels obtained by supplementation with high doses of cholecalciferol had any relation with higher VDR expression and higher LL-37 production, with the purpose of understanding if higher LL-37 levels had any influence on the clinical response and the prognosis in critically ill patients with severe pneumonia caused by SARS-CoV-2.

## 2. Materials and Methods

### 2.1. Selection of Patients, Procedure and Study Design

Participants: We performed a one-center, randomized, not-blind study that included 207 patients admitted to a polyvalent intensive care unit with severe SARS-CoV-2 pneumonia. Patients were recruited from November 2020 to December 2021 and were randomized according to the order of entry into the ICU to three groups of critically ill patients, to whom high (500 MU), moderate (3 MU/day), or no cholecalciferol was given.

Procedure: A total of 66 patients received 500 MU of cholecalciferol in the first 48 h, 72 received 3 MU of cholecalciferol/day during the hospital stay, and 69 received no supplementary cholecalciferol.

Arterial hypertension, obesity, and diabetes mellitus, among other comorbidities, were studied as well as concomitant corticosteroids and other therapies with immunomodulatory effects, to exclude any relationship with clinical course.

From the first study, we realized that only the group of patients supplemented with 500 MU of cholecalciferol had a significant increase in 25vitD levels compared to the others.

To consult the protocol, assess Supplementary Materials S1.

The protocol and consent forms had been previously approved by the hospital ethics committee. Written informed consent or deferred consent was obtained from all patients or their legal surrogates.

We reported our results in a previous paper awaiting publication [56].

Study design: Based on these previous results, we reclassified the patient cohort, initially divided into three groups according to the cholecalciferol doses, into two new groups: patients supplemented with 500 MU of cholecalciferol vs. all the others.

VDR gene expression in monocytes was measured on admission and on the seventh day for the three initial groups.

A post hoc analysis was made where patients supplemented with higher doses of cholecalciferol were included in one group and all other patients in another.

Several laboratory parameters were evaluated from admission to the 7th day of hospitalization, including blood cell count, inflammation markers, and evolution of possible target organ injuries, and safety parameters including serum creatinine, calcium, and phosphorous concentrations.

Clinical and laboratory data were analyzed in patients with and without a significant increase in serum 25vitD levels, after cholecalciferol supplementation or not. LL-37 was measured on admission and on the 3rd day and correlated with cholecalciferol therapy and patient evolution during hospitalization.

### 2.2. Blood Sample Analysis

25vitD

Quantitative determination of 25vitD was obtained by competitive immunoassay using an Atellica IM Analyzer, SIEMENS Healthineers. Plasma levels of 1.25vitD were analyzed by chemiluminescent immunoassay (CLIA), LIASON XL, DiaSorin Inc; prealbumin was analyzed by nephelometry; albumin by colorimetry; C-reactive protein and transferrin by immunoturbidimetry; and ferritin by chemiluminescence.

LL-37

For LL-37 quantification, the samples were cleared by centrifugation at 10,000× *g* for 5 min and the concentration of LL37 was used to quantify LL-37 in ICU patients. A sandwich ELISA was performed with a Human Antibacterial Protein LL-37 ELISA Kit (Abbexa, Cambridge, UK) following the manufacturer’s protocol. In brief, LL-37 standards and patient plasma were added to 96-well plates pre-coated with an anti-LL-37 antibody. A biotin-conjugated reagent was added to the wells and incubated, followed by the addition of the HRP-conjugated reagent. Unbound conjugates were removed using the provided wash buffer at each stage. TMB substrate was used to quantify the HRP enzymatic reaction. Optical density was measured spectrophotometrically at 450 nm.

### 2.3. VDR Gene Expression

Whole blood was diluted with phosphate buffer saline (PBS), pH 7.4, at a 1:1 *v*/*v* ratio. To a SepMate™-50 tube (StemCell Technologies, Vancouver, BC, Canada), 15 mL of Ficoll^®^ Paque Plus (Cytiva, Marlborough, MA, USA) was added, and then 30 mL of the diluted blood was carefully transferred into the tube. The tubes were centrifuged for 10 min at 1200× *g*. Both the plasma and the PBMCs ring were collected into separate tubes. The plasma tube was centrifuged, aliquoted into smaller tubes, and stored at −80 °C. The periferical blood mononuclear cells (PBMCs) were washed three times with PBS and finally resuspended with freezing media (90% fetal bovine serum (FBS) and 10% dimethyl sulfoxide (DMSO) and stored at −80 °C.

The PBMCs were thawed and washed with complete medium (RPMI-1640 supplemented with L-Glutamine, 10% FBS, and 1% PSA) at 37 °C.

Next, to determine the viability, PBMCs were stained with the Live/Dead Fixable Near-IR Dead Cell Stain Kit (Invitrogen, Waltham, MA, USA) and anti-human CD45 (Cytek Bioscience, Clone HI30) and incubated for 10 min at room temperature (RT), in the dark. After fixation with 1% of formaldehyde (FA) (Sigma-Aldrich, St. Louis, MO, USA), viable cell counting was performed in a Cytek Aurora 4L (Cytek Biosciences, Fremont, CA, USA) using SpectroFlo software v3.1. The patients were chosen based on the viability of cells previously determined by flow cytometry. Samples from patients without cholecalciferol supplementation (w/o vit. D), with low cholecalciferol supplementation (Low vit. D), and with high cholecalciferol supplementation (High vit. D), were further divided into two groups: ICU admission (ADM) and 7th day (7th). For each group, 4 participants were pooled. Cell hashing was performed to distinguish each participant from each group. The cell hashing labels each sample with an antibody conjugated to a unique barcode that recognizes CD298 and β2-Microglobulin (Biolegend, San Diego, CA, USA). The pooled samples, after washing, were first incubated with FcR block (Miltenyi Biotec, North Rhine-Westphalia, Germany) and then with TotalSeq™-A Human Universal Cocktail, V1.0 (Biolegend). After the incubation, the cells were washed three times with PBS/BSA and counted, as before, to ensure the same number on each group’s cell input. For the scRNA-seq, the Chromium Next GEM Single Cell 3′ Kit v3.1 (10x Genomics, Pleasanton, CA, USA) was used precisely following the manufacturer’s protocol. All groups were sequenced on Illumina NovaSeq 6000 S4 in one lane following the PE150 strategy. To enable sample demultiplexing, each group was attributed a unique index.

### 2.4. Organ Failure Definitions

To quantify organ failure, an adaptation of the SOFA score was used (0—no failure; 1—organ failure):

For hemodynamic failure: need for vasopressor (0 no vasopressor, 1 vasopressor)

For respiratory failure: PaO_2_/FiO_2_ (0 > 200, 1 < 200 or need for mechanical ventilatory support)

For hematological failure: (0 platelets > 100 × 10^3^/mm^3^, 1 platelets < 100 × 10^3^/mm^3^)

For renal failure: (0 creatinine < 2 mg/dL, 1 creatinine > 2 mg/dL, and/or dialysis needs

For liver failure: total bilirubin (0 total bilirubin < 2 mg/dL, total bilirubin > 2 mg/dL or INR > 2

The total failing organ score was obtained from the sum of failures (0—no failure; 1—organ failure), based on SOFA (above).

### 2.5. Severity Scores

The Simplified Acute Physiology Score (SAPS II) [57] and the Acute Physiology and Chronic Health Evaluation (APACHE II) [58] are conventional disease severity scores and predict the mortality probability of critical care patients.

These scores are made of physiological and disease-related variables, evaluated during the first 24 h in UCI. The worst physiological variables are collected, and a score corresponding to a mortality probability based on the severity of illness is obtained. APACHE II can vary between 0 and 71, and SAPSII between 0 and 163.

### 2.6. Statistical Analysis

The data distribution was tested with the Kolmogorov–Smirnov test for normality. Results were presented as means and standard deviations if normally distributed, or as medians and interquartile ranges (IQR) if non-normally distributed. Categorical variables were presented as frequencies and percentages.

For comparisons between categorical variables, the chi-squared test and the Fisher’s exact test were used.

For comparisons between numeric variables and categorical variables, we used the two-sample unpaired Wilcoxon test (to compare 2 groups) and the Kruskal–Wallis test (to compare more than 2 groups). A *p*-value < 0.05 was considered statistically significant.

When a statistically significant difference was found, we performed a binomial logistical regression analysis and reported odds ratio (OR) with 95% confidence intervals.

A binomial logistic regression was performed to find the risk factors independently related to mortality, and a multivariate logistic regression of mortality outcomes was adjusted for the previously determined independent variables. The Receiver Operating Characteristic (ROC) curve was generated, and the corresponding area under the curve (AUC) was determined to evaluate the accuracy of both models. Additionally, the positive predictive value (PPV) and negative predictive value (NPV) were reported. All the statistical analyses were performed with SPSS version 26.0 (SPSS Inc. Chicago, IL, USA) and RStudio Team (2022) (RStudio: Integrated Development Environment for R. RStudio, PBC, Boston, MA, USA; URL http://www.rstudio.com/ accessed on 3 March 2024).

## 3. Results

Based on our previous study [56], we divided our patients into two groups: those with a significant increase in median serum in 25vitD levels (66 patients who received 500 MU of cholecalciferol in the first 48 h) and those without a significant median increase in 25vitD levels (141 patients that received 3 MU of cholecalciferol/day during their hospital stay or no supplementary cholecalciferol).

The demographic characteristics of patients included in the study are shown in Table 1.

The majority of patients were Caucasian (87.4%) and male (70.5%). Comorbidities and mortality-predicting scores were similar between groups.

Considering SAPS II and APACHE II scoring can vary between 0 and 163 or 0 and 71, respectively, the scores of our patients could suggest less severe patients. However, severe SARS-CoV-2 patients admitted to the ICU cannot be considered mildly ill patients. The fact that they have only one very ill system, the pulmonary system, with some other less severe alterations in other systems, makes these conventional disease severity scores less valid for these particular patients [59]. In this study, these two scores are tools that allow us to confirm how homogenous our groups of patients are (Table 1).

There was a significant difference in 25vitD levels between the two groups on the third (median of 12.5 in patients supplemented with 500 MU of cholecalciferol versus median of 10.3 in the other patients, *p* = 0.002) and seventh (median of 15.6 in patients supplemented with 500 MU of cholecalciferol versus median of 9.2 in the other patients, *p* < 0.001) days but no significant difference between groups in LL-37 levels on admission or on the third day (Table 2).

There was no significant correlation between 25vitD levels on admission and clinical evolution, namely hospital LOS, time in the ICU, ventilation, or need for vasopressors (Table 3(a)).

Patients supplemented with 500 MU of cholecalciferol showed a better course in the ICU, with a significant correlation to evolution factors (Table 3(b)) on the 3rd day: fewer days in the ICU, less hemodynamic failure with lower need for vasopressors, shorter periods of invasive ventilation, and lower need for renal replacement therapy (RRT). On the 3rd day, we found a negative correlation between 25vitD levels and ICU days (r = −0.313, *p* = 0.017), IMV days (r = −0.388, *p* = 0.003), pronation days (r = −0.386, *p* = 0.003), vasopressor days (r = −0.337, *p* = 0.010), or RRT days (r = −0.285, *p* = 0.021).

These correlations were not found in patients without a significant increase in 25vitD levels, or if found, they were weaker.

On the 3rd day, in the other group (3 MU/day or no supplementation), in which 25vitD levels had not a significant increase, there was only a weak negative correlation with IMV days (r = −0.188, *p* = 0.040) and pronation days (r = −0.200, *p* = 0.028).

In terms of prognosis, in our initial analysis using logistic regression, we observed that the LL-37 on admission had a significant negative association with hospital mortality (*p* = 0.045, Table 4) and UCI mortality (*p* = 0.004, Table 5). For every ug/mL increase in LL-37, there is a reduction of around 1.12% in-hospital mortality (Table 4) and a reduction of around 1.42% in ICU mortality (Table 5). We proceeded to adjust the model to comorbidity variables (Table 6 and Table 7).

Not being able to adjust for all variables, we choose the most relevant ones. After adjustment, associations between LL-37 and hospital or ICU mortality lose statistical significance). LL-37 showed a near significant negative association with hospital and even more with ICU mortality. Obesity was associated with a 66% reduction in the odds of hospital mortality and almost a 70% reduction in the odds of ICU mortality. COPD patients had a 4,6 times higher chance of dying in the hospital and an even higher chance of dying in the ICU (5,5 chance) compared with patients without COPD. On the contrary, patients with heart failure had a higher chance of dying in the ward (Table 6) than in the ICU (Table 7) compared with patients without heart failure. Patients with dyslipidemia had an increased chance of dying in the ICU or in the ward, between three and four times higher than patients without (Table 6 and Table 7).

**Figure 1:**
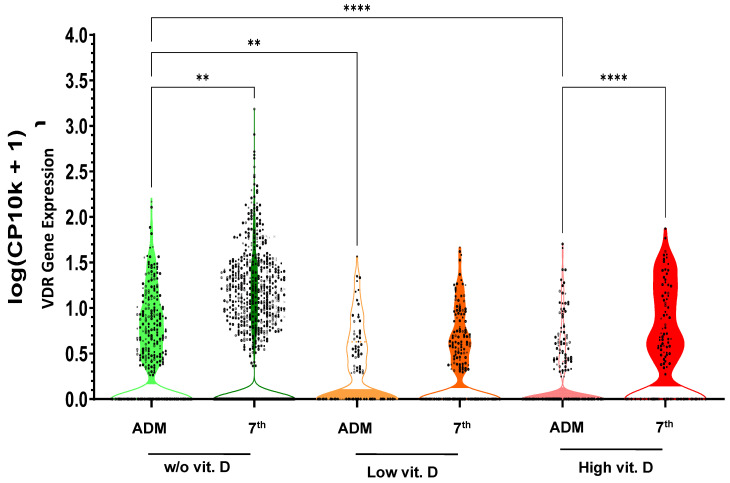
**VDR gene expression in monocytes in COVID-19 ICU patients.** Violin plot displaying the distribution of VDR gene expression (log-transformed counts per 10,000 cells + 1) in monocytes across different conditions: without vitamin D supplementation (**w/o vit.D**), with low (**low vit.D**), or high (**High vit.D**) vitamin D supplementation. Each condition is further divided into admission (**ADM**) and 7th day **(7th)** in the ICU. The statistical significance of pairwise comparisons between conditions is annotated using the Tukey HSD test (** *p* < 0.01, **** *p* < 0.0001).

The AUC shows a fair discriminatory ability of both models in the prediction of mortality. ROC curves (presented in Figure 1) were computed and the area under the curve was 0.747 for ICU and 0.744 for hospital and 60-day mortality. Sensitivity was 66%, specificity was 73.4%, positive predictive value was 15% and negative predictive value was 51.6% for ICU mortality; sensitivity was 56.9%, specificity was 84.2%, positive predictive value was 17.9%, and negative predictive value was 39.6% for hospital mortality. For 60-day mortality, sensitivity was 55.8%, specificity was 84%, positive predictive value was 18.7%, and negative predictive value was 39.6%.

LL-37 also had a prognostic value for 60-day mortality, with a reduction of around 1.24% in 60-day mortality for each ug/mL increase in LL-37 on admission (Table 8).

After adjustment, patients with COPD, dyslipidemia, and heart failure had a 3.7 to 4.5 higher chance of dying within 60 days, compared with patients without these diseases (Table 9).

Regarding immunomodulatory therapy, non-significant associations were found for corticoid use. However, on simple logistic regression, remdesivir use was significantly associated with ICU (OR: 0.23, 95%IC: 0.053–0.68, *p* = 0.018), hospital (OR: 0.20, 95%IC: 0.04–0.61, *p* = 0.0125), and 60-day mortality (OR: 0.28, 95%IC: 0.08–0.757, *p* = 0.028). However, in the multiple logistic regression model, for all three outcome variables, remdesivir use lost statistical significance.

APACHE II and SAPS II scores are not good severity discriminatory scores with which to distinguish disease severity in patients with severe SARS-CoV-2 pneumonia. These patients are usually very ill but with few organ failures, mainly respiratory, and systematically low APACHE II and SAPS II scores. There was not a significant correlation between SAPS II and 25vitD levels, nor was there a significant difference between the groups, which proves the great homogeneity of the sample. There is no correlation between 25vitD levels on admission and the tools for severity assessment and prognosis usually used in the ICU.

However, as referred previously, we observed a negative correlation between 25vitD and severity markers on day 3, mainly in the group with a high dose of cholecalciferol supplementation (Table 3(b)), which is the only group where 25vitD levels had a significant increase after cholecalciferol supplementation. This significant correlation also exists for some severity markers on the 7^th^ day, but it loses strength over time (Table 3(c)).

On the other hand, LL-37 showed no significant increase after cholecalciferol administration, not even in the group with a significant 25vitD increase.

VDR gene expression in monocytes in the three initial groups of patients was evaluated on admission and on the third day in the ICU (group supplemented with 500 MU of cholecalciferol on the first 48 h, group supplemented with 3 MU of cholecalciferol/day during hospital stay and group with no supplementary cholecalciferol).

From VDR gene expression in monocytes, we realized that the group of patients supplemented with a high dose of cholecalciferol had a much more significant increase in monocyte VDR gene expression from admission to the third day than the other two groups, although the group supplemented with 3 MU of cholecalciferol/day had a lower but still significant increase in VDR gene expression. The group not supplemented had a significant decrease in VDR gene expression (Figure 1).

An increase in VDR gene expression leads to a higher VDR protein in cells, enhancing the body’s ability to respond to vitamin D. VDR mediates 1.25vitD action in calcium/phosphate translocation between tissues, and also influences immune, neural, epithelial, and endocrine systems [60,61].

It is possible that the increase in VDR gene expression represents an increase in functional VDR, increasing anti-inflammatory and immunomodulatory functions, particularly important in this group of patients.

## 4. Discussion

In our previous study [56], we observed a statistically significant difference in median levels of 25vitD between groups on the third day in the ICU (D3) (*p* = 0.008) and on the seventh (D7) (*p* < 0.001), but not on admission before supplementation, with higher median levels of 25vitD in the 500 MU supplemented group at D3 (Z = 1047, *p* < 0.001) and D7 (Z = 781, *p* < 0.001). In the other groups, including the group supplemented with 3 MU/day, a decrease in median serum concentration of 25vitD over time was found in Table 3 in [56]. From these results, we concluded that only patients submitted to supplementation with a higher dose of cholecalciferol had a significant increase in vitD levels. In the present study, we evaluated if the group of patients with a significant increase in 25vitD levels had an increase in LL-37 production, with the purpose of understanding if an increase in LL-37 levels had any influence on the clinical response and prognosis in critically ill patients with severe pneumonia caused by SARS-CoV-2. There was no relation between cholecalciferol supplementation and LL-37 increase.

Despite no significant mortality reduction in critically ill patients with severe pneumonia caused by SARS-CoV-2 supplemented with high-dose cholecalciferol, a failure of the efficacy of cholecalciferol administration is not conclusive. Higher 25vitD and higher LL-37 levels on admission may both be protective. There was a significant decrease in severity markers.

Even higher doses of cholecalciferol may be needed to show significant mortality results. Several factors for cholecalciferol increased need can be proposed: some kind of resistance to cholecalciferol related to age or clinical condition may exist, or even an increased catabolism related to other therapies or to the catabolic and inflammatory state. There were no side effects, analytical complications, or other safety concerns related to high doses of cholecalciferol administration, making it possible to test higher doses in future trials and explore both boosted and continuous administration.

As patients were treated in a single ICU and all had the same diagnosis, they were all submitted to an identical clinical approach, eliminating some of the bias that can occur in multicenter studies and related to different pathologies.

Unfortunately, when we decided to supplement our patients with two different doses of cholecalciferol, we divided our 207 patients’ samples into three groups, and our sample size became smaller. We are convinced that a greater sample size could have had different and more conclusive results.

Although there were no significant differences in 25vitD and LL-37 levels on admission, and in LL-37 levels between our two groups on the 3rd day, we cannot ignore that patients supplemented with 500 MU had a higher 25vitD on admission and on the 3rd day, and also higher LL-37 levels in both situations, although these differences were not significant when compared with the other group. In this regard, we cannot be certain that higher levels before illness are not protective either. Mardi A Crane-Godreau and colleagues [62] also concluded that a weakened response against SARS-CoV-2 could be related to reduced LL-37.

Higher LL-37 on admission had a significant negative association with mortality, but this significance was lost after adjustment for comorbidities. Perhaps a larger sample would be able to show significant results and the benefit of higher LL37 levels on admission.

According to our work, the significant difference in 25vitD levels between the patients supplemented with higher doses of cholecalciferol and the others on the third and seventh days may suggest that only supplementation with higher doses of cholecalciferol can contribute to a significant increase in 25vitD levels in patients with severe SARS-CoV-2 pneumonia and to a better prognosis. Igor and colleagues [63] reported an increase in vitD levels as well, but with only a single inferior dose of cholecalciferol; however, they could not show any difference in terms of length of stay, which could be related to the need for larger doses for beneficial effect, as they only supplemented with 200 MU once.

We had already made a preliminary study with supplementation with a higher dose of cholecalciferol, 500 MU, in a group of 72 patients and we found an association with lower all-cause ICU, intrahospital, and total mortality. These data have been accepted for publication [64].

Supplementation with cholecalciferol was shown to be safe, with no significant side effects. Higher doses of cholecalciferol and also a larger number of supplemented patients may be necessary to obtain significant results.

In vitro [19] and in vivo [65] studies suggest that vitamin D is able to upregulate LL-37 levels, contributing to the innate immune system.

A more thorough understanding of the interactions between vitamin D and LL-37, as well as their relationship with VDR gene expression and immunomodulation, is essential. The current targets for blood levels of 25(OH)D and LL-37 in critically ill patients in particular require reassessment and potential reestablishment.

Further studies are needed to evaluate the influence of baseline vitamin D levels on clinical outcomes and prognosis, as elevated levels of LL-37 and 25(OH)D may be associated with improved patient outcomes.

Investigations focusing on cholecalciferol supplementation in critically ill patients are warranted. Higher doses of cholecalciferol may enhance VDR gene expression and contribute to better prognoses without significant adverse effects.

The inclusion of a homogeneous population of critically ill patients with a single admission pathology—severe SARS-CoV-2 pneumonia—and standardized treatment protocols presented a unique opportunity to minimize the bias typically observed in critical care studies. Such bias often arises from heterogeneous pathologies, varied treatments, and divergent patient trajectories. However, dividing the sample compromised the statistical power of the study. While the study was conducted safely, the question of whether higher doses of supplementation are more efficacious remains unresolved. The investigation was limited to a single center and vitamin D levels in individuals are influenced by seasonal and geographical factors. Therefore, the study could not ensure the complete exclusion of potential confounders. Broader, multi-center collaborations would have strengthened the findings and reduced bias underscoring the importance of global efforts in this field.

## 5. Conclusions

The results obtained highlight the potential of vitamin D to enhance clinical outcomes in critical care patients. Nevertheless, larger, multi-center, and more diverse studies are required to determine optimal supplementation strategies and dosages. Such efforts will facilitate the development of evidence-based guidelines to maximize the therapeutic efficacy of vitamin D in critical care settings.

## Figures and Tables

**Table 1:** Baseline characteristics of patients.

Variable	Total (*n* = 207)	500 MU Vitamin D	3 MU/day or No Vitamin D	*p*-Value
		(n = 66)	(n = 141)	
Age—years (sd)	57.7 (13.5)	56.9 (12.6)	58.1 (13.8)	0.561
Female sex—n (%)	61(29.5)	16 (24.2)	45(31.9)	0.259
Race—n (%)				0.98
Black	20 (9.7)	6 (9.1)	14 (9.9)	
White	181 (87.4)	58(87.9)	123 (87.2)	
Asian	6 (2.9)	2 (3.0)	4 (2.8)	
Comorbidities—n (%)				
Hypertension	113 (54.6)	31 (47.0)	82 (58.2)	0.132
Diabetes	60 (29.0)	18 (27.3)	42 (29.8)	0.71
Dyslipidemia	51 (29.7)	18 (31.6)	33 (28.7)	0.697
Obesity	85 (41.1)	27 (40.9)	58 (41.1)	0.975
Heart failure	15 (7.2)	4 (6.1)	11 (7.8)	0.653
Chronic kidney disease	11 (5.3)	2 (3.0)	9 (6.4)	0.316
COPD	15 (7.2)	8 (12.1)	7 (5.0)	0.064
Body mass index—mean (SD)	29.0 (5.40)	29.35 (5.48)	28.84 (5.39)	0.555
Remdesivir use—n (%)	32 (15.5)	10 (15.2)	22 (15.6)	0.933
Corticoid use—n (%)	207 (100.0)	66 (100.0))	141 (100.0))	
Dexametasone	201 (97.1)	66 (100.0)	135 (95.7)	0.089
Metilprednisolone	15 (7.2)	3 (4.5)	12 (8.5)	0.305
SOFA score—median (mín-max)	6 (2–12)	4 (2–12)	6 (3–12)	0.336
APACHE II score—mean (SD)	10.3 (5.9)	9.5 (4.5)	10.7 (5.1)	0.418
SAPS II score—mean (SD)	33.1 (11.7)	31.6 (11.0)	33.8 (12.0)	0.93

**Table 2:** 25vitD and LL37 levels between groups.

Variable	Total (n = 207)	500 MU Vitamin D (n = 66)	3 MU/day or No Vitamin D (n = 141)	*p*-Value
25 vit D admission—median (IQR)	15.7 (12)	16.2 (13.0)	15.7 (12.2)	0.650
25 vit D 3rd day—median (IQR)	14.2 (10.2)	15.2 (12.5)	14.0 (10.3)	0.002
25 vit D 7th day—median (IQR)	14.3 (10.1)	19.2 (15.6)	13.0 (9.2)	<0.001
LL 37 admission—median (IQR)	16.1 (31.5)	16.9 (34.3)	15.6 (29.8)	0.995
LL 37 3rd day—median (IQR)	833.0 (1744)	1074.5 (2315.5)	779.5 (1630.8)	0.685
LL 37 variation—median (IQR)	799.9 (1714.7)	1046.2 (2289.7)	750.1(1630.3)	0.381

**Table 3:** (a) Correlations * with 25vitD on admission; (b) correlations * with 25vitD on 3rd day; (c) correlations * with 25vitD on 7th day.

**(a)**			
**Cholecalciferol** **Administration**	**Total (n = 207)**	**500 MU Vitamin D** **(n = 66)**	**3 MU/day or No Vitamin D (n = 141)**
**Spearman ρ, *p*-Value**			
Hospital LOS	n.s.	n.s.	n.s.
ICU days	n.s.	n.s.	n.s.
IMV days	n.s.	n.s.	n.s.
Pronation days	n.s.	n.s.	n.s.
Vasopressor days	n.s.	n.s.	n.s.
RRT days	n.s.	n.s.	n.s.
VitD25 1st day	n.a.	n.a.	n.a.
VitD25 3rd day	0.792, <0.001	0.713, <0.001	0.830, <0.001
VitD25 7th day	0.633, <0.001	0.490, <0.001	0.817, <0.001
LL37 adm	n.s.	n.s.	n.s.
LL37 3rd day	n.s.	n.s.	n.s.
LL37 var	n.s.	n.s.	n.s.
**(b)**			
**Cholecalciferol** **Administration**	**Total (n = 207)**	**500 MU Vitamin D** **(n = 66)**	**3 MU/day or No Vitamin D (n = 141)**
**Spearman ρ, *p*-Value**			
Hospital LOS	n.s.	n.s.	n.s.
ICU days	n.s.	−0.313, 0.017	n.s.
IMV days	−0.274, <0.001	−0.388, 0.003	−0.188, 0.040
Pronation days	−0.282, <0.001	−0.386, 0.003	−0.200, 0.028
Vasopressor days	−0.241, 0.001	−0.337, 0.010	n.s.
RRT days	n.s.	−0.285, 0.030	n.s.
VitD25 1st day	0.792, <0.001	0.713, <0.001	0.870, <0.001
VitD25 3rd day	n.a.	n.a.	n.a.
VitD25 7th day	0.808, <0.001	0.673, <0.001	0.886, <0.001
LL37 adm	n.s.	n.s.	n.s.
LL37 3rd day	n.s.	n.s.	n.s.
LL37 var	n.s.	n.s.	n.s.
**(c)**			
**Cholecalciferol** **Administration**	**Total (n = 207)**	**500 MU Vitamin D** **(n = 66)**	**3 MU/day or No Vitamin D (n = 141)**
**Spearman ρ, *p*-Value**			
Hospital LOS	n.s.	n.s.	n.s.
ICU days	n.s.	n.s.	n.s.
IMV days	n.s.	−0.287, 0.050	n.s.
Pronation days	−0.252, 0.003	−0.309, 0.035	n.s.
Vasopressor days	−0.185, 0.033	n.s.	n.s.
RRT days	n.s.	n.s.	n.s.
VitD25 1st day	0.633, <0.001	0.490, <0.001	0.817, <0.001
VitD25 3rd day	0.808, <0.001	0.673, <0.001	0.886, <0.001
VitD25 7th day	n.a.	n.s.	n.a.
LL37 adm	n.s.	0.304, 0.040	n.s.
LL37 3rd day	n.s.	n.s.	n.s.
LL37 var	n.s.	n.s.	n.s.

* Spearman correlation.

**Table 4:** Logistic regression for hospital mortality.

	Estimate	S.E.	z Value	Sig.	OR	CI
Constant	−0.5683	0.201	−2.828	0.0047	0.5664644	0.3818–0.8407
LL37 adm	−0.0112	0.006	−2.008	0.0446	0.9888198	0.9767–0.9984

**Table 5:** Logistic regression for ICU mortality.

	Estimate	S.E.	z Value	Sig.	OR	CI
Constant	−0.6006	0.209	−2.879	0.0039	0.548461	0.3641–0.8258
LL37 adm	−0.0142	0.006	−2.228	0.0259	0.985909	0.9722–0.9967

**Table 6:** Logistic regression adjusted for comorbidities.

	Estimate	S.E.	z Value	Sig.	OR	CI
Constant	−0.94656	0.302	−3.137	0.0017	0.3880753	0.2111–0.6938
LL37 adm	−0.00862	0.005	−1.632	0.1026	0.9914181	0.9795–1.0005
Obesity	−1.07408	0.404	−2.654	0.0079	0.3416129	0.1491–0.7358
Dyslipidemia	1.38486	0.397	3.489	0.0005	3.9942490	1.8562–8.8619
COPD	1.53278	0.648	2.367	0.0179	4.6310377	1.3065–17.2138
Heart Failure	1.55322	0.645	2.407	0.0161	4.7266648	1.3505–17.8403

Variable(s) entered: LL37 adm: LL37 on admission, Heart failure, COPD (Chronic Obstructive Pulmonary Disease), Obesity, Dyslipidemia (colest T > 240 e/ou TG > 200 mg/dL). OR: Odds ratio; S.E.: standard error; CI: confidence interval.

**Table 7:** Logistic regression adjusted for comorbidities.

	Estimate	S.E.	z Value	Sig.	OR	CI
Constant	−0.98036	0.312	−3.144	0.0017	0.3751705	0.2000–0.6841
LL37 adm	−0.01065	0.006	−1.765	0.0775	0.9894068	0.9757–0.9994
Obesity	−1.17622	0.422	−2.786	0.0053	0.3084418	0.1293–06840
Dyslipidemia	1.35001	0.407	3.321	0.0009	3.8574730	1.7580–8.7212
COPD	1.69928	0.654	2.600	0.0093	5.4700249	1.5316–20.6558
Heart Failure	1.32540	0.643	2.061	0.0392	3.7637009	1.0483–13.6922

Variable(s) entered: LL37 adm: LL37 on admission, Heart failure, COPD (Chronic Obstructive Pulmonary Disease), Obesity, Dyslipidemia (colest T > 240 e/ou TG > 200 mg/dL). OR: Odds ratio; S.E.: standard error; CI: confidence interval.

**Table 8:** Logistic regression for 60-day mortality.

	Estimate	S.E.	z Value	Sig.	OR	CI
Constant	−0.4939	0.201	−2.460	0.0139	0.6102230	0.4117–0.9057
LL37 adm	−0.0124	0.006	−2.164	0.0304	0.9876442	0.9752–0.9974

**Table 9:** Logistic regression adjusted for comorbidities.

	Estimate	S.E.	z Value	Sig.	OR	CI
Constant	−0.86080	0.298	−3.144	0.0039	0.4228224	0.2319–0.7526
LL37 adm	−0.00914	0.005	−1.704	0.0884	0.9909059	0.9788–1.0001
Obesity	−1.11025	0.403	−2.756	0.0058	0.3294769	0.1443–0.7070
Dyslipidemia	1.33194	0.395	3.373	0.0007	3.7883878	1.7664–8.3656
COPD	1.48361	0.646	2.296	0.0217	4.4088255	1.2469–16.352
Heart Failure	1.52313	0.644	2.354	0.0181	4.5865784	1.3132–17.2852

Variable(s) entered: LL37 adm: LL37 on admission, Heart failure, COPD (Chronic Obstructive Pulmonary Disease), Obesity, Dyslipidemia (colest T > 240 e/ou TG > 200 mg/dL). OR: Odds ratio; S.E.: standard error; CI: confidence interval.

## Data Availability

Data available on request.

## Supplementary Materials

The following supporting information can be downloaded at: https://www.mdpi.com/article/10.3390/nu17030540/s1, Supplementary Materials S1: Prospective Study on The Immunomodulatory Role of Vitamin D in Patients with Severe SARS-Cov-2 Pneumonia Admitted to a Polyvalent Intensive Care Unit. The Protocol.

## Abbreviations

The following abbreviations are used in this manuscript:	
AMPs	Antimicrobial peptides
APACHE	Acute Physiology and Chronic Health Evaluation
AUC	Area under the curve
CI	Confidence interval
Colest T	Total cholesterol
COPD	Chronic Obstructive Pulmonary Disease
D3	Third day
D7	Seventh day
HIV	Human immunodeficiency virus
ICU	Intensive care unit
IMV	Invasive mechanical ventilation
IQR	Interquartile range
LL-37	Leucine-leucine-37
LL37 adm	Leucine-leucine-37 on admission
LL37 var	LL37 1st day–LL37 3rd day
LOS	Length of stay
Max	Maximum
Min	Minimum
MU	Thousand units
n.a.	Non-applicable
NPV	Negative predictive value
n.s.	Non-significant
OR	Odds ratio
PPV	Positive predictive value
ROC	Receiver Operating Characteristic
RRT	Renal replacement therapy
SAPS	Simplified Acute Physiology Score
SARS-CoV-2	Severe acute respiratory syndrome coronavirus 2
SD	Standard deviation
SE	Standard error
SOFA	Sequential organ failure assessment
TG	Triglycerides
ug	Micrograms
1.25vitD	1.25-dihydroxycholecalciferol
25vitD	25 hydroxyvitamin D
VitD	Vitamin D
VDR	Vitamin D receptor
