# Supplementary material for: The Immunomodulatory Activity of High Doses of Vitamin D in Critical Care Patients with Severe SARS-CoV-2 Pneumonia—A Randomized Controlled Trial
Source: Nutrients. 2025 Jan 31;17(3):540. doi: 10.3390/nu17030540 (PMC11820001; doi:10.3390/nu17030540)
Supplement: Supplementary file 1 [file nutrients-17-00540-s001.zip › nutrients-3434469-supplementary.pdf]

Supplement S1

Prospective Study on The Immunomodulatory Role of Vitamin D in Patients with Severe SARS-Cov-2 Pneumonia Admitted to a Polyvalent Intensive Care Unit. The Protocol

Ana Moura Gonçalves[1], João Gonçalves[2], António Marinho[3]

[1] *Department of Intensive Care Medicine - Hospital Beatriz Ângelo*
[2] *Faculty of Pharmacy – University of Lisbon iMed – Research Institute of Medicines*
[3] *Faculty of Medicine, Instituto de Ciências Biomédicas Abel Salazar - University of Porto*

Objectives
The main objective of the study is to understand the role of vitamin D in patients with severe SARS Cov2 pneumonia
Assess the impact that different doses of vitamin D have on:
- the clinical course and prognosis of patients
- the evolution of routine laboratory parameters
- the immunological parameters
- the activation and expression of the RVD
- the expression of the LL 37 peptide

Hypothesis
Will moderate or high doses of vitamin D play a protective role, activating the immune response in critically ill patients hospitalized for severe SARS-CoV-2 pneumonia?
Could different doses of vitamin D have different effects on clinical course, severity and prognosis of these patients?

Proposed studies, sample to be studied and methodologies to be adopted
A nonblinded randomized controlled trial was conducted in patients with severe SARS-CoV-2 pneumonia admitted to the ICU.

<u>Proposed studies:</u>
1- Study the effect of Cholecalciferol on the clinical evolution of patients with severe SARS Cov2 pneumonia.
2- Study the effect of Cholecalciferol at different doses on serum levels of LL-37 and its activity
3- Study the effect of Cholecalciferol in different doses on serum levels of VDR gene expression

<u>Selection of patients and study design:</u>
Initially the study population included 221 critically ill patients admitted to the intensive care unit for severe SARS Cov2 pneumonia, with or without associated ARDS and without any other reason for admission to the ICU. Patients could have other comorbidities as long as their decompensation was not the reason of ICU admission.

Exclusion criteria: Patients aged under 18 years, pregnant women, patients with an estimated ICU or hospital stay of less than 48 hours, surgical patients, patients with prolonged decompensated heart failure, patients with shock caused by a condition other than respiratory infection, patients with known stage 4 chronic kidney disease, patients with severe hypercalcemia, with advanced stage neoplasms or other chronic pathologies in progress, namely hematological, and patients with no possibility of early enteral feeding.
Three critically ill patients randomized groups were established (randomized according to the order of entry into the ICU, without prior knowledge of the allocation key) to whom cholecalciferol was given or not in moderate or high doses (nonblinded, randomized, controlled study).
The ICU admission of the patient was a responsibility of senior Intensivists, without knowledge of the allocation key.
Patients were placed further into the study, sequentially:
2 patients in the cholecalciferol-free arm,
next 2 patients in the cholecalciferol 250 000U/day arm (12.5 mL of 0.5 mg/ml cholecalciferol oral solution, orally or by nasogastric tube) for 2 days,
and the next 2 patients in the 2000U/day cholecalciferol arm (3 drops of 0.5 mg/ml cholecalciferol oral solution, orally or by nasogastric tube) during the ICU stay and remaining hospitalization.

This allocation was made by the physicians responsible for patient admission, after consulting the allocation arm.

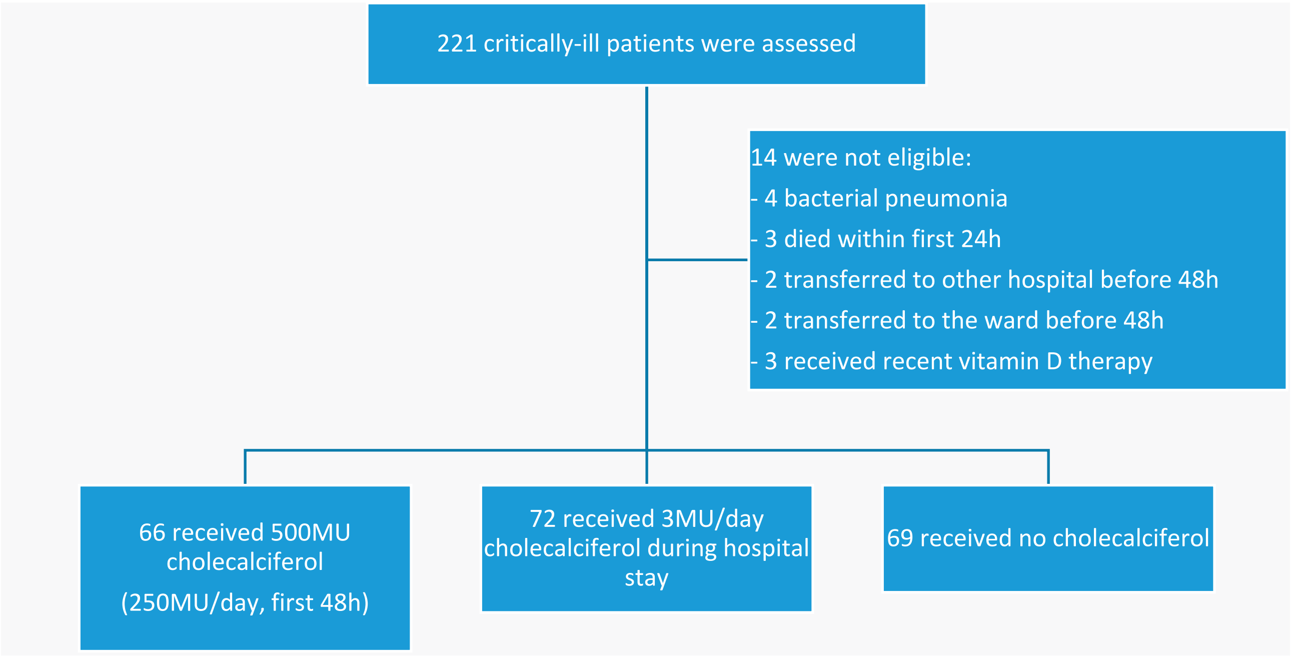

It was intended to evaluate several laboratory parameters from admission to the 7th day of hospitalization:

- blood count, inflammation markers,

- evolution of possible target organ injuries

- serum 25-hydroxyvitamin D (25vitD) levels,

- vitamin D receptor (VDR) gene expression,

- LL37 levels

- the immune response of leukocytes and cytokines.

It was also intended to correlate 25vitD and the other blood results with the clinical evolution and prognosis of patients infected with severe SARS-Cov-2 pneumoniae, depending on the presence or absence of therapy with cholecalciferol.

<u>Organ failure definitions</u>

Adapted from SOFA score (0- no failure; 1- organ failure):
Hemodynamic failure: need for vasopressor (0 no vasopressor, 1 vasopressor)
Respiratory failure; PaO2/FiO2 (0 >200, 1 <200 or need for mechanical ventilatory support)
Hematological failure (0 platelets > 100x103/mm3, 1 platelets < 100x103/mm3)
Renal failure (0 creatinine < 2 mg/dL, 1 creatinine > 2 mg/dL and/or dialysis needs
Liver failure: total bilirubin (0 total bilirrubin < 2 mg/dL, total bilirrubin > 2 mg/dL or INR >2
Total failing organ score was obtained from the sum of failures (0- no failure; 1- organ failure), based on SOFA (above).

The main comorbidities were also considered, namely arterial hypertension, obesity, diabetes mellitus, among others, as well as concomitant therapies with an immunomodulatory effect (corticosteroids and other immunomodulatory therapy), to exclude any relationship with clinical evolution.
Patients were included during 1 year.

- **Blood sample profile evaluated in the hospital, in all patients with COVID 19 infection admitted to a level III ICU:**

  On admission:

- Complete blood count - Coagulation with prothrombin time (TP) and activated partial thromboplastin time (APTT) - D-dimers - Urea - Creatinine - Ionogram with sodium (Na), potassium (K), Chlorine (Cl), magnesium (Mg) and phosphorus (P) - Arterial blood gases (GSA) - Calcium - Aspartate transaminase (AST) - Alanine transaminase (ALT) - Alkaline phosphatase (FA) - Gamma glutamyl transpeptidase (GGT) - Total and direct bilirubin - Lactate dehydrogenase (LDH) - Creatine kinase (CK) - Myoglobin - Troponin - Brain natriuretic peptide (NTproBNP) - Sedimentation rate (VS) - C-reactive protein (PCR) - Procalcitonin (PCT) - Total proteins - Albumin - Ferritin - Fibrinogen - 1.25 vitamin D - 25 vitamin D - Parathyroid hormone (PTH) - Total cholesterol and high density lipoprotein (HDL) - Triglycerides - B12, folate - Troponin - myoglobin - IL-6

  Daily evaluation

- Complete blood count - Coagulation with TP and APTT - D-dimers - GSA - Urea -Creatinine - Ionogram with Na, K, Cl, Mg and P-AST -ALT -FA - GGT - Direct direct and total bilirubin - LDH - CK - Myoglobin - Troponin - NTproBNP - PCR - PCT

  Assessment on admission, at 72h and 7th to 10th day

- Albumin - Ferritin - Fibrinogen

- VS - 1.25 vitamin D - 25 vitamin D - PTH – Calcium

- **Analytical profile evaluated at the Faculty of Pharmacy in all patients with COVID 19 infection admitted to a level III ICU (on admission, at 72h and on the 7th day):**

- Quantification of the SARS-Cov-2 anti-Spike IgM, IgG and IgA response
- Quantification of the viral neutralizing capacity of the antiviral antibody response.
- Quantification of anti-SARS-CoV-2 cellular response
- Interleukins: IL-6, MCP-1 (CCL2), G-CSF, IFN-a2, IL-2, IFN-gamma, IL-7, IL-1RA, IL-8 (CXCL8), TNF-alpha, IP -10, (CXCL10), MIP-1a (CCL3), RANTES (CCL5), IL-10, IL-13, GM-CSF, IL-1ß, IL-5, sCD25 (IL-2Ra), IL-4, VEGF, IL-17A, IL-18, APRIL, MIP-1ß (CCL4), IL-15, IL-12p70.
- Proteins/peptides: LL-37 and vitamin D receptor (VDR) gene expression on lymphocytes and antigen presenting cells.
- Sequencing of total RNA of cell transcripts in single-cell by massive sequencing (NGS) in peripheral blood lymphocytes.
- Evaluation of proteomics of cell markers in immunological populations (154 markers -CD86, CD274, TNFRSF14, PVR, NECTIN2, CD47, CD48, CD40, CD40LG, CD52, CD3D, CD8A, NCAM1,CD19, CD33, ITGAX, HLA-A, PTPRC, IL3RA, CD7, ITGA6, CCR4, CD4, CD44, CD14, FCGR3A, IL2RA, PTPRC, PDCD1,TIGIT, MS4A1, NCR1, PECAM1, PDPN, MCAM, IGHM, CD5, CXCR3, CCR5, FCGR2A, CCR6, CXCR5, ITGAE, CD69, SELL, KLRB1, CTLA4, LAG3, KLRG1, CD27, LAMP1, FAS, TNFRSF4, HLA-DRA, CD1C, ITGAM, FCGR1A, THBD, KLRK1, CR1, B3GAT1, BTLA, ICOS, ICOSLG, CD58, ENTPD1, CX3CR1, CD24, CR2, ITGAL, CD79B, CD244, SIGLEC1, ITGB7, TNFRSF13C, GP1BB, ICAM1, SELP, IFNGR1, CCR2, IL2RB, TNFRSF13B, FCER1A, ITGA2B, TNFRSF9, SPN, CD163, CD83, ANPEP, CD2, CD226, ITGB1, CLEC4C, ITGA2, ITGB3, CD81, CD55, IGHD, ITGB2, CD28, CD38, IL7R, PTPRC, CD22, TFRC, DPP4, CSF1R, CD63, NRP1, CD36, SIRPA, CD72, KIR2DL1, CD93, CD200, ITGA1, ITGA4, NT5E, CD9, TREM1, LAIR1, OLR1, KIR3DL1, CD109, F3, SLAMF7, CD99, CLEC12A, CD151, SLAMF6, CLEC1B, KLRD1, IGHE, SLAMF1, SELPLG, CD84, IGKC, LILRB1, FCER2, SIGLEC7, ADGRG1, CD82, KLRF1, CSF2RB, CD74, CSF2RA, CD37, CD101, HLA-DRA, C5AR1)

Blood sample analysis

Quantitative determination of 25vitD was obtained by competitive immunoassay, Atellica IM Analyzer, SIEMENS healthineers; plasma levels of 1,25vitD were analyzed by chemiluminescent immunoassay (CLIA), LIASON XL, DiaSorin Inc; prealbumin was analyzed by nephelometry; albumin by colorimetry; C- Reactive protein and transferrin by immunoturbidimetry and ferritin by chemiluminescence.

LL37 quantification

For LL37 quantification, the samples were cleared by centrifugation at 10,000 x g for 5 minutes and the concentration of LL37 was used to quantify LL-37 in ICU patients. A sandwich ELISA was performed with a Human Antibacterial Protein LL-37 ELISA Kit (Abbexa, Cambridge, United Kingdom) following the manufacturer's protocol. In brief, LL-37 standards and patient plasma were added to 96-well plates pre-coated with an anti-LL-37 antibody. A biotin-conjugated reagent was added to the wells and incubated, followed by the addition of the HRP-conjugated reagent. Unbound conjugates were removed using the provided wash buffer at each stage. TMB substrate was used to quantify the HRP enzymatic reaction. Optical density was measured spectrophotometrically at 450 nm.

All samples were collected and properly packaged for transport to the Molecular Pathogenesis Center at the Faculty of Pharmacy of the University of Lisbon.
Serum 25vitD will be measured on admission, on third and seventh hospital day and correlated with organ failure score, acute phase markers, ICU length of stay, days on ventilator, need for vasopressors, infectious complications, need for renal function substitution and hospital mortality.

Discussion

With this study, we hope to understand the role of vitamin D in the immune response in critically ill patients with severe pneumonia caused by SARS Cov2.
Could vitamin D in moderate or high doses have a protective role, activating the immune response of patients with SARS Cov2 infection, reducing aggressiveness and worse clinical evolution?
Does it influence immunological complications, reducing cytokines storm in SARS Cov2 infection?
Could different doses of vitamin D have different effects on clinical evolution, disease severity and prognosis of critically ill patients?
If vitamin D benefits are proven, it is expected that the health benefits will far outweigh the costs, and a more comprehensive study in critically ill patients with septic shock from other causes can be tried.
The authors pretend to answer these questions with the study described in this protocol.

Ethics Statement and Acknowledgments

The study has the approval of the Ethics Committee and the Scientific Committee of the Beatriz Ângelo hospital, where blood samples are collected. To participate in the study, all patients or their legal representatives must give their authorization through an informed consent document.

Author Conflict of Interest

There is no conflict of interest for all participant authors.
